# Malaria vectors in the Democratic Republic of the Congo: the mechanisms that confer insecticide resistance in *Anopheles gambiae* and *Anopheles funestus*

**DOI:** 10.1186/s12936-017-2099-y

**Published:** 2017-11-07

**Authors:** Luisa Nardini, Richard H. Hunt, Yael L. Dahan-Moss, Nanette Christie, Riann N. Christian, Maureen Coetzee, Lizette L. Koekemoer

**Affiliations:** 1 Wits Research Institute for Malaria, School of Pathology, Faculty of Health Sciences, University of the Witwatersrand, Johannesburg, 2000 South Africa; 2 Centre for Emerging, Zoonotic & Parasitic Diseases, National Institute for Communicable Diseases, Johannesburg, 2131 South Africa; 3 Department of Genetics, Forestry and Agricultural Biotechnology Institute, University of Pretoria, Pretoria, 0028 South Africa

**Keywords:** *Anopheles funestus*, *Anopheles gambiae*, Deltamethrin resistance, *kdr*, Metabolic resistance, P450s, GST

## Abstract

**Background:**

The Democratic Republic of the Congo (DRC) is characterized as a holoendemic malaria area with the main vectors being *Anopheles funestus* and members of the *Anopheles gambiae* complex. Due to political instability and socio-economic challenges in the region, knowledge of insecticide resistance status and resistance mechanisms in these vectors is limited. Mosquitoes were collected from a mining site in the north-eastern part of the country and, following identification, were subjected to extensive testing for the target-site and biochemical basis of resistance. Quantitative real-time PCR was used to assess a suite of 10 genes frequently involved in pyrethroid and dichlorodiphenyltrichloroethane (DDT) resistance in *An. gambiae* females and males. In *An. funestus*, gene expression microarray analysis was carried out on female mosquitoes.

**Results:**

In both species, deltamethrin resistance was recorded along with high resistance and suspected resistance to DDT in *An. gambiae* and *An. funestus,* respectively. A total of 85% of *An. gambiae* carried the *kdr* mutations as either homozygous resistant (RR) (L1014S, L1014F or both) or heterozygous (RS), however only 3% carried the *rdl* mutant allele (RS) and no *ace*-*1* mutations were recorded. Synergist assays indicated a strong role for P450s in deltamethrin resistance in both species. In *An. gambiae*, analysis of transcription levels showed that the glutathione-S-transferase, *GSTS1*-*2*, produced the highest fold change in expression (7.6-fold in females and 31-fold in males) followed by *GSTE2*, thioredoxin peroxidase (*TPX2*), and cytochrome oxidases (*CYP6M2* and *CYP6P1*). All other genes tested produced fold change values below 2. Microarray analysis revealed significant over-transcription of cuticular proteins as well as *CYP6M7*, *CYP6P9a* and *CYP6P9b* in insecticide resistant *An. funestus*.

**Conclusions:**

These data show that high levels of deltamethrin resistance in the main malaria vector species, conferred by enzymatic detoxification, are present in the DRC.

**Electronic supplementary material:**

The online version of this article (10.1186/s12936-017-2099-y) contains supplementary material, which is available to authorized users.

## Background

The severe burden of malaria in Africa is related to a number of factors including the presence of well-adapted vectors, human activities, such as deforestation, agriculture and urbanization, and poor healthcare due to socio-economic and political factors [1, 2]. Malaria transmission is further enhanced through “occupational activities” such as mining that bring humans and vectors into contact [1]. Widespread drug resistance in *Plasmodium* parasites and insecticide resistance in the vectors exacerbate the problem [3]. All these factors are relevant in the Democratic Republic of the Congo (DRC) which is characterized by high transmission (i.e. more than 1 case per 1000 population per year) of *Plasmodium falciparum* by the highly efficient vectors, *Anopheles funestus*, *Anopheles gambiae* and *Anopheles coluzzii* [3, 4]. Vector control in the DRC is largely based on the use of long-lasting insecticide treated bed nets (LLINs) [3, 4], and to a lesser extent, indoor residual spraying (IRS) which is limited to areas where mining operations are present [5].

A number of mechanisms confer resistance to a small pool of insecticides and for this reason, insecticide resistance management is both challenging and essential. Pyrethroids, organochlorines, carbamates and organophosphates are the only four classes of insecticide approved for use in vector control [6]. In particular, there has been a strong reliance on the pyrethroids as they are the only class of insecticide approved for treating bed nets, which have been central to reducing malaria prevalence. The pyrethroids are fast acting, inexpensive and demonstrate low mammalian and environmental toxicity. All four classes have been used for IRS.

Dichlorodiphenyltrichloroethane (DDT) is highly effective against many malaria vector populations and is relatively cost effective, however due to safety concerns, it is allowed for use in IRS programmes only. Given that DDT and pyrethroid resistance is widespread, interest in the carbamates and organophosphates for IRS is growing (see Refs. [7–10]) and in 2013, carbamates and organophosphates were used by 12 and 13 countries, respectively [11]. However, the use of the latter two is disadvantageous from a cost point of view, and more recently, resistance to these insecticides has been reported [12, 13].

Insecticide resistance is largely based on target-site insensitivity due to the occurrence of mutations and/or through enhanced enzymatic detoxification. Altered acetylcholinesterase, knockdown resistance (*kdr*) and mutations in the ‘resistance to dieldrin’ gene are all examples of target-site mutations. Knockdown resistance refers to point mutations in the voltage gated sodium channel (VGSC), the target of DDT and pyrethroids. Three mutations in the VGSC are well described, they are L1014S, L1014F and more recently, N1575Y [14]. The West African mutation results in a change from leucine to phenylalanine (L1014F) of the S6 segment [15] while the East African version occurs at the same codon but is a leucine to serine mutation (L1014S) [16]. Although the naming of the mutations as East and West was based initially on where they were found in Africa, studies have since shown that the mutations are not restricted to these areas. In addition, both mutations have been found in one population [17, 18]. A number of studies have shown that *kdr* is often present in the DDT- and pyrethroid-resistant phenotypes, but the use of synergists have shown that metabolic based mechanisms may also be responsible for resistance, even if the mutation is present [19–21]. Furthermore, many studies that evaluate the presence of *kdr* do not evaluate the presence of metabolic mechanisms through the use of synergists, quantitative real-time PCR or biochemical methods [22–24]. The mutations have been found in *An. gambiae, An. coluzzii* and *Anopheles arabiensis* [25, 26].

The *ace*-*1* ^*R*^ mutation is a glycine to serine substitution at position 119 in acetylcholinesterase that is related to carbamate and organophosphate resistance [27]. Given that pyrethroid and DDT resistance are so common, interest in carbamates and organophosphates as alternatives for use in IRS has increased and monitoring for the *ace*-*1* ^*R*^ mutation has become important [13]. The mutation has been found in *An. gambiae*, *An. coluzzii* and *An. arabiensis* vector populations in Ghana [13], Burkina Faso [28, 29], Côte d’Ivoire [30] and at low frequency in Benin [31]. With the use of dieldrin banned for public health, the presence of mutations in the γ-amino butyric acid (GABA) receptor is often ignored [32]. However, it is interesting that where these mutations occur, they remain established, and they have the potential to confer cross-resistance to other insecticides (both agricultural and public health) with the same target site [18, 33].

Metabolic detoxification involves three large enzyme families: the cytochrome P450s, the glutathione S-transferases (GSTs) and the esterases. Knowledge of the identity and functions of the enzymes that confer resistance is an important aspect of vector control so that resistance can be managed, the potential for cross-resistance can be reduced, and novel insecticide targets can be investigated. It has been shown that some enzymes are able to directly metabolize particular insecticides. For example, *GSTe2* [34] and *CYP6M2* [35] are able to metabolize DDT and pyrethroids, and *CYP6Z1* is able to metabolize DDT [36] however in numerous studies many detoxification enzymes are highly over-transcribed in the resistant phenotype, suggesting that multiple enzymes may play a role in the resistance phenotype. In *An. gambiae* and *An. arabiensis*, genes such as *CYP6M2*, *CYP6P3* [20, 37, 38] and *CYP4G16* [39, 40] are frequently found over-transcribed, while in *An. funestus*, *CYP6P9a*, *CYP6P9b*, *GSTe2* [34, 41] and *CYP6M7* [42] have been implicated in metabolic detoxification.

Often, vector status and insecticide resistance monitoring, when conducted, are not sufficiently comprehensive in many countries. This imposes limitations on successful decision-making in national and local vector management programmes. This project aims to contribute to the knowledge of vector composition, insecticide resistance status and mechanisms conferring resistance in vectors from north-eastern DRC.

## Methods

### Entomological surveys and mosquito collections

Entomological surveys and collections were conducted at the Kibali Gold Mine in the Moto goldfields, north-eastern DRC (N03°08.846′E29°36.548′) in March/April 2011 and July 2012. Adult collections were done by manual aspiration in and around mine houses and structures, as well as in the homes and structures of nearby communities. According to initial field identifications using morphological keys [43], *An. gambiae* complex and *An. funestus* group were collected. A sub-set of wild caught females of each group were used for standard WHO insecticide susceptibility bioassays while the remainder of the females (2011: *An. gambiae* complex = 101, *An funestus* group = 101; 2012: *An. gambiae* complex = 88, *An. funestus* group = 94) were brought back to the insectary at the National Institute for Communicable Diseases (NICD) in Johannesburg for further analysis. For this purpose, females were separated into glass vials, blood fed and provided with moist filter paper for oviposition. Each family produced from a female was given a number for identification purposes.

### Species identification

Mosquitoes were identified to species level by polymerase chain reaction (PCR) for the *An. gambiae* complex [44], *An. coluzzii* (previously M molecular form) or *An. gambiae s.s.* (previously S form) [45], and the *An. funestus* group [46].

### *Plasmodium falciparum* sporozoite detection

Enzyme-linked immunosorbent assay (ELISA) was used to detect the presence of *P. falciparum* [47, 48]. The head and thorax of individual mosquitoes were used, and in all assays, positive and negative controls were included. Reactions were assayed spectrophotometrically at 405 nm (Ascent Multiskan RC vl. 5.0, Genesis version 3.03, Labsystems) and all positive samples were assayed a second time.

### WHO bioassays and synergist exposures

Where possible, field collected adults (males and females) of each species were exposed in the field to DDT (4%), deltamethrin (0.75%), propoxur (0.1%), fenitrothion (1%), malathion (5%), bendiocarb (0.1%) and dieldrin (4%), according to standard WHO procedures [49]. In each case, mosquitoes were exposed to a particular insecticide for 1 h (2 h for fenitrothion), and sample sizes per exposure varied from 12 to 30 mosquitoes per tube depending on mosquito numbers. Mosquitoes were provided with a 10% sugar solution ad libitum and after 24 h, mortality was recorded. During the 2012 collections, too few *An. gambiae* were collected for field-based bioassays. For this reason, live specimens were returned to the laboratory and F1 progeny were used for insecticide exposures to DDT, deltamethrin, propoxur, fenitrothion, malathion, bendiocarb and dieldrin.

**Fig. 1:**
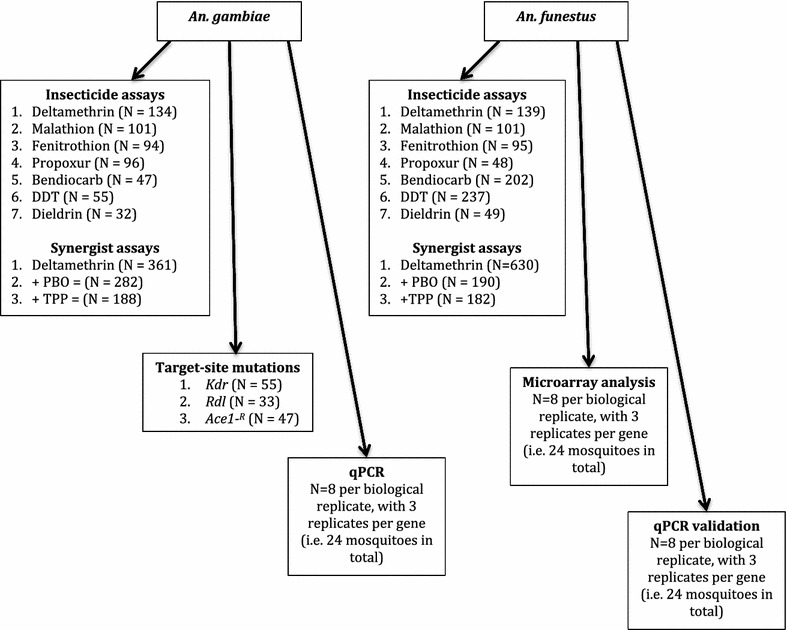
The number of specimens tested in each assay are outlined for clarity, for the 2012 field season

In the second survey (2012), F1 offspring from individual families generated from both *An. funestus* and *An. gambiae* were used for synergist assays prior to deltamethrin exposure. These exposures comprised 1–4 day old males and females. Exposures were carried out as described above, however the number of replicate exposures varied for each family depending on the number of offspring available. These exposures were prepared in conjunction with synergist assays where the monooxygenase inhibitor, piperonyl butoxide (PBO) and the esterase inhibiter, triphenylphosphate (TPP) were used to synergize the F1 offspring of *An. gambiae* (PBO N = 282; TPP N = 188) and *An. funestus* (PBO N = 190; TPP N = 182) prior to insecticide exposure. Between 5 and 20 adults were exposed, per replicate, to 4% PBO or 10% TPP for an hour depending on mosquito availability, and then immediately exposed to 0.75% deltamethrin for an hour before being returned to a holding tube. Mortality was recorded after 24 h. Insecticide exposure versus synergist plus insecticide exposure were analysed using the Student’s *t*-test. An outline of the key assays is provided in Fig. 1.

### Molecular evaluation of resistance mechanisms in *Anopheles gambiae s.s.*

Both target site mutations and metabolic mechanisms were evaluated for the 2012 field season. Target site molecular markers were evaluated for the following alleles: *Rdl* (A296G), *kdr* (L1014S and L1014F), *ace*-*1* ^*R*^ (G119S). Known metabolic genes (*GSTS1*-*2*, *GSTE2*, *TPX2*, *CYP6M2*, *CYP6P1*, *CYP6AG2*, *CYP4G16*, *CYP9L1*, *CYP6Z1* and *SOD1*) were evaluated in F1 progeny using quantitative real-time PCR (qPCR).

### Detection of *kdr*

Polymerase chain reaction (PCR) followed by direct sequencing of the relevant gene was performed in order to test for the presence of *kdr* east and west mutations in specimens assayed against DDT in the *An. gambiae* field samples. DNA was extracted from both survivors (N = 47) and mortalities (N = 8), stored on silica gel, using the *prep*GEM Insect Kit (ZyGEM). Each PCR reaction (containing 2.5ul 10 × PCR Buffer, 1.5 μl MgCl_2_ [25 mM], 2.5 μl dNTP’s [2.5 mM each], 3 μl each of AGD1 and AGD2 primers [10 µM], 0.2 μl Takara Taq and 11.3 μl nuclease free water) was subjected to the following cycling conditions: 94 °C/2 min (min), (94 °C/30 s [sec], 50 °C/30 s, 72 °C/30 s) × 40 cycles, and a final extension step at 72 °C for 5 min. The primers, AGD1 (5′ ATA GAT TCC CCG ACC ATG 3′) and AGD2 (5′ AGA CAA GGA TGA TGA ACC 3′), span the region containing the mutations [15]. Results were viewed by electrophoresis and column purified amplicons were sequenced in both directions by Macrogen (Sanger sequencing using the ABI 3730XL DNA Sequencing system). Sequences were aligned using DNASTAR (Lasergene Megalign 2007) software and were screened for the *kdr*. Positive and negative controls in the form of laboratory strains were included in order to assist with interpretation.

### Detection of “resistance to dieldrin” mutation

The presence of the A296G mutation was assayed using TaqMan^®^ technology according to the method of Bass et al. [50]. A mutant specific probe and wild type probe were included in the assay as well as two standard forward and reverse primers. Both survivors (N = 1) and mortalities (N = 32) were assayed and DNA was extracted using the *prep*GEM Insect Kit (ZyGEM). The resistant laboratory strain COGS (*An. gambiae s.s.* colonized from the Republic of the Congo in 2009 [18]) was used as a positive (homozygous resistant (RR) and heterozygous (RS) control while the susceptible SUA strain (*An. coluzzii* from Liberia) was used as a homozygous susceptible control (SS).

### Detections of ACE mutation

PCR of the *ace*-*1* ^*R*^ mutation and subsequent digestion was performed in order to detect the G119S mutation in the acetylcholinesterase neurotransmitter [27]. DNA was extracted using the *prep*Gem Insect Kit (ZyGEM) from mosquitoes that both survived (N = 22) and died (N = 25) following exposure to bendiocarb (0.1%). Amplification of the *ace*-*1* ^*R*^ gene to produce a 541 base pair product was performed using the forward Ex3Agdir (5′ GAT CGT GGA CAC CGT GTT CG 3′) and reverse Ex3Agrev (5′ AGG ATG GCC CGC TGG AAC AG 3′) primers under the following conditions: 94 °C/3 min, 35 cycles of [94 °C/30 s, 62 °C/30 s, 72 °C/20 s] and a final extension at 72 °C/5 min. Each reaction comprised 2.5 μl 10 × PCR Buffer, 2.5 μl dNTPs [2.5 mM each], 1.5 μl MgCl_2_ [25 mM], 4 μl of each primer at a concentration of 2 μM, 0.2 μl Takara Taq and 12.8 μl nuclease-free H_2_O. Digestion using *Alu I* (Promega) restriction enzyme was carried out and visualized with positive and negative controls by agarose gel electrophoresis. Restriction enzyme digestion produces two fragments of 403 and 138 bp in the SS genotype, while three fragments (of 253, 150 and 138 bp) characterize the RR genotype.

### RNA extraction

RNA was extracted from 1 to 5 day old *An. gambiae* F1 progeny using TRI^®^ Reagent Solution (Sigma-Aldrich) [51]. For each of the three biological repeats, 2 mosquitoes from four different families were used (N = 8), and for each biological repeat, four new families were used. A susceptible laboratory strain of *An. coluzzii*, SUA, was used as a control and three corresponding biological repeats were prepared. The RNA samples were reverse transcribed into cDNA using the QuantiTect^®^ Reverse Transcription Kit (Qiagen) according to supplier instructions.

### Real-time quantitative PCR (qPCR)

Real-time qPCR was used to evaluate transcription of 10 genes commonly known to, or previously implicated in playing a role in insecticide resistance against pyrethroids. Reference gene (RG) selection was performed as specified by the minimum information for publication of quantitative real-time PCR experiments [52] where the expression in 4 potential reference genes, namely ribosomal protein L19 (*RPL19*), ribosomal protein S7 (*RPS7*), *β*-*actin* and *18S* was evaluated and analysed using the MS Excel add-in, NormFinder [53]. For *An. gambiae*, *RPL19* and *β*-*actin* showed the most stable transcription in both males and females. The over-transcription of the genes of interest was measured relative to these two RGs. Each qPCR reaction was set up as follows: 12.5 μl IQ™ SYBR super-mix (Bio-Rad), 4 μl primer (concentration optimized for each gene), 1 μl cDNA (100 ng/μl) and nuclease free water to a volume of 25 µl. PCR was carried out using the Bio-Rad CFX96™ Real-Time PCR Detection System with the following cycling conditions: 93 °C/3 min, followed by 35 cycles of [94 °C/20 s, optimized annealing temperature/25 s, 72 °C/30 s] with a single extension at 72 °C/10 min. and a final melt. Standard curves were prepared by 2-fold dilutions of cDNA derived from the wild mosquitoes. For each gene, three biological repeats were assessed, and for each biological repeat, three technical repeats were included for each reaction. Data were analysed using the Pfaffl [54] method. Initially, column-purified PCR product for each gene of interest was sent to Macrogen for Sanger sequencing in both directions in order to confirm (in addition to melt curve analysis) that the correct product was amplified in each case. The genes evaluated in male and female *An. gambiae* were *GSTS1*-*2*, *GSTE2*, *TPX2*, *CYP6M2*, *CYP6P1*, *CYP6AG2*, *CYP4G16*, *CYP9L1*, *CYP6Z1* and *SOD1*. Primer sequences are provided in Table 1.

**Table 1:** Primer sequences used for relative quantification of genes linked to the insecticide resistance phenotype in *Anopheles gambiae*

Gene	Sequence (5′–3′)	Primer conc. ( μM)	Annealing temperature ( °C)	Amplicon size (bp)	Reference
GSTS1-2 F GSTS1-2 R	GCT GTC TTA CGG CAA CCT TC CCA CGG TGT CAA TCA TCA AG	3	58.3	212	–
GSTe2 F	CAT TTG AAG CCG GAA TTT GT	3	60	123	–
GSTe2 R	TTT GCC ATA CTT CGT CAC CA				
TPX2 F TPX2 R	GGA TGT TTG TGG GGA ATA CG TGT GCG ATT AGC CTC CTC TT	3	52	165	[20]
CYP6M2 F CYP6M2 R	CAT GAC ACA AAC CGA CAA GG GGT GAG GAG AGT CGA CGA AG	3.5	52	235	[20]
CYP6P1 F	CGC GCA GGT GTT TAT CTT TT	3	60	199	–
CYP6P1 R	GTT CAC CAC CTG TCC GAG AT				
CYP6AG2 F CYP6AG2 R	TTG TGC TGC CGT ACT ATT CG TAC TAT CGC CCG TCT CAC CT	3	60	200	[20]
CYP4G16 F CYP4G16 R	CAG ACC GTC CAG CCA CAT TC GCG AAC GAG CAA TTA TAG GTA CTG	3	60	108	[20]
CYP9L1 F CYP9L1 R	AGA TAA TGT ATT CTT TCG CTA TGG GCT CTT CTC GCT CTT GAA C	3	56.3	188	[20]
CYP6Z1 F	TTA CAT TCA CAC TGC ACG AG	3	57	146	–
CYP6Z1 R	CTT CAC GCA CAA ATC CAG AT				
β-actin F β-actin R	ACC AAG AGC CTG AAG CAC CGA GCA CGA CAC ACT ATA TAC	N/A	–	123	[83]
RPL19 F RPL19 R	CCA ACT CGC GAC AAA ACA TTC ACC GGC TTC TTG ATG ATC AGA	N/A	–	61	[20]

The gene and primer names are designated as *F* forward primer and *R* reverse primer, and primer concentration (conc.), annealing temperature, amplicon size and primer citation are included where relevant. Annealing temperature of the reference genes *β*-*actin* and *RPL19* were the same as that for each test gene (depicted as N/A)

### Evaluation of resistance mechanisms in *Anopheles funestus*

As no *kdr* has been found in *An. funestus* to date, this was not evaluated. The presence of *ace*-*1* ^*R*^ or *rdl* mutants was not included as *An. funestus* from this population were susceptible to bendiocarb and dieldrin, respectively.

### Preparation of microarrays and analysis

RNA was extracted from 1 to 5 day old *An. funestus* F1 progeny [51]. For each of the three biological repeats, two mosquitoes from four different families were used (N = 8), and for each biological repeat, four different families were used. A susceptible laboratory strain, FANG (*An. funestus* colonized from Angola in 2002), was used as a control and three corresponding biological repeats were prepared. RNA was extracted using the TRI^®^ Reagent Solution (Sigma-Aldrich) according to supplier instruction, with a DNase treatment included (RNase-Free DNase Set, Qiagen). The quality and quantity of the RNA was assessed using a NanoDrop 2000 Spectrophotometer (Thermo Fisher Scientific Inc.). Four microarrays were prepared using the Agilent 4 × 44 K platform (AMADID 048099)—one for each biological repeat, and a dye swap to account for bias in dye binding. Each array consisted of 33,022 *An. funestus* specific probes available in Genbank and 1417 Agilent control features. A total of 303 nucleotides belonged to the cytochrome P450 class, 11 to the GST class, 23 to the esterases class and a large number of other insecticide resistant genes.

Labelling was done using the Low Input Quick Amp Labelling Kit (Agilent Technologies) according to manufacturer’s instructions. A starting quantity of 1 μg RNA was incorporated into each labelling reaction. Labelled targets were purified using the RNEasy^®^ Mini Kit (Qiagen) and successful labelling was evaluated using a NanoDrop Spectrophotometer (Thermo Fisher Scientific Inc.). Hybridization was performed provided dye incorporation was above 0.1 pmol/μl and cRNA yield above 36.2 ng/μl. In addition, specific activity was determined as (concentration of Cy3/Cy5)/(concentration of RNA)*1000 = pmol Cy3 or Cy5 per ug RNA. Specific activity above 8 pmol Cy3 or Cy5 per μg RNA, with a yield above 825 ng was acceptable. Hybridization was performed using the Agilent Gene Expression Hybridization Kit (Agilent Technologies) and was incubated at 65 °C/18 h. After this period, slides were washed and scanned using the GenePix 4000B scanner (Molecular Devices, USA) where the photomultiplier tube (PMT) settings were adjusted to give a pixel ratio of approximately 1. Spot quality and background intensities were examined and corrected using GenePix Pro 6.0 software (Axon Instruments, USA). Saturated features were excluded from analysis (the PMT gain was set at maximum and minimum limits of 63,000).

Gene expression data were analysed using the limma (linear models for microarray data) package version 2.12.0 (Bioconductor) [55] in R version 2.8.0 [56]. Data were normalized (‘within-array’ global loess normalization and ‘between-array’ Aquantile normalization), and linear models were fitted in order to contrast resistant *An. funestus* expression values with expression values of a susceptible strain. Differentially expressed probes were defined as those with a fold-change greater or equal to 2 and with adjusted *p*-value ≤ 0.05 [*p*-values associated with the moderated *t*-test were corrected for multiple testing based on the false discovery rate (FDR)]. Batch Entrez [57] was used to retrieve the nucleotide sequences linked to accession numbers of all probes on the microarray (25,404 sequences were retrieved in FASTA format). Blast2GO [58] was used to functionally annotate the probe sequences, and for gene ontology (GO) enrichment of over- and under-transcribed gene sets. The *p*-values for the GO-enrichment analysis were calculated using Fisher’s Exact Test and an adjustment for multiple testing was performed to control the FDR. In order to obtain additional annotation information, basic local alignment search tool (BLAST) [59] searches were carried out against all PEST and *Aedes aegypti* transcripts (FASTA files were downloaded from Vectorbase [60]). For each *An. funestus* query sequence, the top PEST and *Aedes aegypti* nucleotide BLAST hit was recorded if the e-value cutoff was less than 1e-05 (see Additional file 1).

### Real-time quantitative PCR (qPCR)

Real-time PCR was used for validation of *An. funestus* microarray data as described above. Reference genes used for *An. funestus* were *RPL19* and *RPS7* as they were found to be the most stable RGs. The genes selected for microarray validation in *CYP6M7* and *CYP6P9b*, and primer details are summarized in Table 2.

**Table 2:** Primer sequences used for relative quantification of genes linked to the insecticide resistance phenotype in *Anopheles funestus*

Gene	Sequence (5′–3′)	Primer conc. ( μM)	Annealing temperature ( °C)	Amplicon size (bp)	Reference
CYP6P9b F CYP6P9b R	CAG CGC GTA CAC CAG ATT GTG TAA TTA CAC CTT TTC TAC CTT CAA GTA ATT ACC CGC	3	60	97	[41]
CYP6M7 F CYP6M7 R	CGT TGT ATG AGC TGG CGT TA GTG CAT CTC CAT GAC AGC AT	3	60	116	[41]
RPL19 F RPL19 R	CCA ACT CGC GAC AAA ACA TTC ACC GGC TTC TTG ATG ATC AGA	3	–	61	[20]
RPS7 F RPS7 R	TTA CTG CTG TGT ACG ATG CC GAT GGT GGT CTG CTG GTT C	3	–	135	[84]

The gene and primer names are designated as *F* forward primer and *R* reverse primer, and primer concentration (conc.), annealing temperature, amplicon size and primer citation are included where relevant

## Results

### Species identification, WHO susceptibility tests and sporozoite detection

#### *Anopheles gambiae* complex

In 2011, 580 mosquitoes of unknown age were used for field-based bioassays. Although low levels of resistance will be missed by using wild adults, it does however provide valuable insight as to which resistance mechanisms needed to be targeted when F1 progeny were available [18]. During the 2012 survey, F1 progeny were used for bioassays (N = 559) as the number of *An. gambiae* collected in the field were too few for meaningful bioassay tests to be conducted. Species identification was confirmed by PCR as *An. gambiae s.s*. (previously S molecular form). No *An. coluzzii* (M form) or *An. arabiensis* were identified. In 2011, the field bioassays indicated resistance to deltamethrin (51% mortality), DDT (60% mortality) and propoxur (86% mortality), with full susceptibility to the organophosphates (Table 3). The 2012 assessment revealed an even higher level of resistance to DDT (15% mortality) while resistance to deltamethrin remained high (44% mortality). In contrast, the mosquitoes reverted to propoxur susceptibility (86% in 2011 versus 100% mortality in 2012) but bendiocarb resistance had developed (97% in 2011 versus 53% mortality in 2012). Full susceptibility to the organophosphates was maintained. An exceptionally high level of *P. falciparum* sporozoite positivity (8.8%) was recorded in 2011.

**Table 3:** Mortality recorded in *Anopheles gambiae s.s*. males and females collected during the 2011 (adults collected in the field) and 2012 field (F1 progeny of field-collected adults) seasons following 1-h insecticide exposures using standard WHO tubes

Insecticide	2011			2012		
	N	% Mortality	Resistance status	N	% Mortality	Resistance status
Deltamethrin (0.75%)	97	51	Resistant	134	44	Resistant
Malathion (5%)	81	100	Susceptible	101	99	Susceptible
Fenitrothion (1%)	112	100	Susceptible	94	99	Susceptible
Propoxur (0.1%)	90	86	Resistant	96	100	Susceptible
Bendiocarb (0.1%)	90	97	Suspected resistance	47	53	Resistant
DDT (4%)	58	60	Resistant	55	15	Resistant
Dieldrin (0.4%)	52	100	Susceptible	32	97	Suspected resistance

Interpretation of resistance status is based on WHO criteria [49]

#### *Anopheles funestus* group

A total 662 *An. funestus* were assayed for insecticide resistance in the field in 2011, while in 2012, a total of 871 *An. funestus* were tested in WHO bioassays. The mosquitoes were confirmed as *An. funestus*, and no other members of the *An. funestus* group were present. In the 2011 season, *An. funestus* were largely susceptible to all insecticides tested, although evidence of emerging deltamethrin resistance was observed with 93% mortality after 1-h exposure (Table 4). Worryingly, full resistance to deltamethrin was observed in 2012 (with 69% mortality) and potential resistance to DDT had emerged (95% mortality). Susceptibility to the organophosphates was reported and maintained in 2011 and 2012. As in the case of *An. gambiae*, an unusually high percentage of mosquitoes (12.2%) were found to be positive for *P. falciparum* in the 2011 field season.

**Table 4:** Mortality recorded in *Anopheles funestus* males and females collected during the 2011 and 2012 field assays following 1-h insecticide exposures using standard WHO tubes

Insecticide	2011			2012		
	N	% Mortality	Resistance status	N	% Mortality	Resistance status
Deltamethrin (0.75%)	100	93	Suspected resistance	139	69	Resistant
Malathion (5%)	88	100	Susceptible	101	100	Susceptible
Fenitrothion (1%)	110	100	Susceptible	95	100	Susceptible
Propoxur (0.1%)	72	97	Suspected resistance	48	100	Susceptible
Bendiocarb (0.1%)	142	98	Susceptible	202	98	Susceptible
DDT (4%)	101	99	Susceptible	237	95	Suspected resistance
Dieldrin (0.4%)	49	100	Susceptible	49	100	Susceptible

Interpretation of resistance status is based on WHO criteria [49]

#### Synergist assays

Use of the monooxygenase specific synergist, PBO, revealed that deltamethrin was largely based on P450 based metabolism with complete reversion to susceptibility after exposure to PBO in *An. funestus*, and almost complete reversion to susceptibility in *An. gambiae* (F1 progeny in both species used for these assays) (Table 5). Synergizing mosquitoes with TPP prior to deltamethrin exposure produced no change in susceptibility to deltamethrin.

**Table 5:** Percentage mortality observed in F1 *Aopheles gambiae* and *An. funestus* males and females following exposure to the synergists (PBO or TPP) and insecticide

	0.75% Deltamethrin (n)	0.75% Deltamethrin + PBO (n)	0.75% Deltamethrin + TPP (n)
*An. gambiae s.s.*	30% (361)^a^	92 ± 15% (282)^b^	28 ± 23% (188)^a^
*An. funestus*	59% (630)^a^	100 ± 0% (190)^b^	51 ± 31% (182)^a^

^a,b^Indicate statistically significant differences determined by Students’ *t*-test between deltamethrin (0.75%) + synergist and deltamethrin (0.75%) only

### Molecular evaluation of resistance mechanisms in *Anopheles gambiae*

#### *Rdl*-mutation

**Fig. 2:**
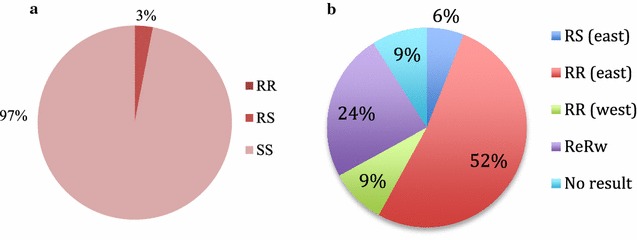
Outcome of mutation assays for **a** *rdl* mutant alleles and **b** *kdr* mutant east African (L1014S) and west African (L1014F) mutant alleles. In part (**a**), 3% represents 1 specimen out of 33. In part (**b**), representative specimen numbers are as follows: RS (east) = 3, RR (east) = 29, RR (west) = 5, ReRw = 13, and 5 specimens failed to amplify (i.e. no result was obtained)

Only one mosquito survived dieldrin exposure in 2012, and was analysed for the presence of the *rdl* mutation. Those that survived exposure (N = 32) were also genotyped. The specimen that survived exposure to dieldrin was heterozygous for the mutation (Fig. 2a). All those that died were homozygous SS.

#### *Kdr*-mutation

Of the 55 specimens tested against DDT, 53% were homozygous resistant for the east mutation (RR_L1014S_), while only 9% were homozygous for the west mutation (RR_L1014F_) (Fig. 2b). Heterozygotes for the east (R_L1014S_S) form were found (6%), but none for the west mutant. A large percentage (24%) of the specimens carried both mutations. The *kdr* status for 9% could not be determined by sequencing or by TaqMan^®^ assays.

#### *Ace*-*1*^*R*^ mutation

There were no *ace*-*1* ^*R*^ mutants detected in any bendiocarb resistant specimens (N = 47) from 2012 collections.

#### Real-time quantitative PCR

**Fig. 3:**
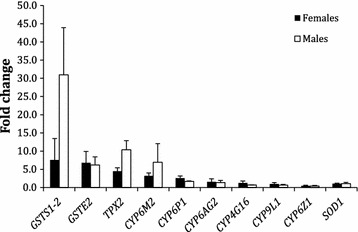
The fold change reported for adult *An. gambiae* male and female specimens from the DRC. Genes that are frequently reported as playing a role in insecticide resistance to pyrethroids were selected for analysis. The fold change (FC) values reported here represent the average FC values measured against two different reference genes (RGs) *RSP7* and *RPL19* (three biological repeats per RG). Bars represent standard deviation

Genes that are frequently reported as playing a role in insecticide resistance to pyrethroids were selected for analysis by real-time quantitative PCR in *An. gambiae* males and females. In males, a greater than 5-fold increase was observed in *GSTS1*-*2*, *GSTe2*, *TPX2* and *CYP6M2* when compared with the selected reference genes (RGs). In females, *GSTS1*-*2* and *GSTe2* showed over-transcription greater than 5-fold, while *TPX2, CYP6M2* and *CYP6P1* were more than 2-fold over-transcribed relative to the selected RGs (Fig. 3).

### Molecular evaluation of resistance mechanisms in *Anopheles funestus*

*Anopheles funestus* gene expression arrays were prepared to evaluate changes in gene expression in resistant female *An. funestus* from the DRC using a susceptible laboratory strain as a reference. According to our criteria for cut-off (FC ≥ 2.0 and adjusted *p*-value ≤ 0.05), a total of 409 and 280 probes were significantly over- and under-transcribed respectively (Additional file 1). Using Blast2GO, enriched gene ontology terms were assigned to the sets of over- and under-transcribed genes. As expected, genes with a variety of functions produced significantly increased transcript abundance, although proteins with oxidoreductase activity, and cuticular proteins predominated (*p*-value < 0.05, FDR < 0.1) as determined by enrichment analyses (Table 6). Amongst the most highly over-transcribed genes were ubiquitin c (EZ916542), a component of the ubiquitin proteolytic system [61], at 8.4-fold over-transcribed; troponin C (EZ915656), an important protein in calcium dependent regulation of muscle contraction [62] at 5.7-fold over-transcribed; and the oxido-reductase enzyme sorbitol dehydrogenase, 8.2-fold over-transcribed (Table 7). Other highly transcribed non-detoxification genes included chymotrypsin 1 (7.3-fold over-transcribed), and a range of cuticle proteins. Five known cytochrome P450s were significantly over-transcribed, namely *CYP6M7* (7.7-fold), *CYP6P9b* (3.3-fold), *CYP6P9a* (2.0-fold) and the duplicated genes, *CYP6P4a* and *CYP6P4b*, at 2.0- and 2.1-fold over-transcribed respectively (Table 8). The DDT-metabolizing *GSTe2* was 2-fold over-transcribed, while a second GST, un-annotated in *An. funestus*, but which mapped to GSTs1 in *An. gambiae* was also significantly over-transcribed (3.6-fold) (Table 8).

**Table 6:** Significantly enriched gene ontology terms (GO) terms, using Blast2Go, of over-transcribed genes in insecticide resistant *Anopheles funestus* relative to susceptible controls

GO Description	GO Type	Adjusted *p*-value (FDR)	Cluster frequency
Oxidoreductase activity	Molecular function	3.0E−06	16/929
Structural constituent of the cuticle	Molecular function	7.7E−06	4/24
Oxidation–reduction process	Biological process	1.7E−05	14/824

**Table 7:** The top 35 over-transcribed genes with available descriptions, in wild insecticide resistant *Anopheles funestus* relative to the susceptible laboratory strain, FANG

Accession number	Log_2_FC	FC	Adjusted p-value (FDR)	Best-hit Blast2Go description	Best-hit PEST descriptions
EZ916542	3.07	8.4	4.6E−04	Ubiquitin c variant 2	Polyubiquitin-B
EZ976930	3.03	8.2	8.2E−05	Cuticular protein 49aa cg30045-pb	CPR113: cuticular protein RR-2 family 113
EZ967106	2.97	7.9	8.1E−05	–	Myosin heavy chain
EZ980273	2.96	7.8	1.4E−02	tbc domain-containing protein kinase-like protein	TBC domain-containing protein kinase-like protein
EZ973782	2.94	7.7	8.2E−05	Cytochrome p450 6a8	CYP6M3: cytochrome P450
EZ915918	2.89	7.4	1.3E−04	Actin	Actin, cytoplasmic
EZ979664	2.86	7.3	8.1E−05	CHYMOTRYPSIN 1	CHYM1: chymotrypsin-1
EZ915406	2.75	6.7	8.1E−05	Sorbitol dehydrogenase	d-xylulose reductase A
EZ918406	2.70	6.5	8.1E−05	Ubiquitin c variant 2	Polyubiquitin
EZ980370	2.53	5.8	5.9E−03	Slow border cells	CCAAT/enhancer binding protein (C/EBP), invertebrate
EZ917907	2.53	5.8	3.7E−04	Flightin	Flightin
EZ919993	2.52	5.8	8.7E−05	–	CPLCA3: cuticular protein 3 in CPLCA family
EZ925592	2.51	5.7	1.2E−04	–	NADH dehydrogenase (ubiquinone) 1 alpha subcomplex 4
EZ915656	2.51	5.7	8.7E−05	Troponin c	Troponin C
EZ917820	2.41	5.3	6.4E−04	–	CPR125: cuticular protein RR-2 family 125
EZ916349	2.37	5.2	9.0E−05	Actin	Actin, cytoplasmic
EZ966655	2.36	5.1	4.4E−04	Transforming growth factor beta regulator 1	Uncharacterized protein
EZ920676	2.30	4.9	6.9E−04		Troponin T, fast skeletal muscle
EZ966156	2.28	4.8	8.8E−03	Muscle lim protein	Muscle LIM protein at 84B
EZ915396	2.24	4.7	1.2E−04	Sorbitol dehydrogenase	d-xylulose reductase A
EZ917325	2.18	4.6	1.2E−04	Regulator of microtubule dynamics protein 1-like	Regulator of microtubule dynamics protein 1-like
EZ920255	2.18	4.5	1.2E−04	–	Muscle LIM protein at 84B
EZ915403	2.15	4.4	1.2E−04	Sorbitol dehydrogenase	d-xylulose reductase A
EZ917889	2.09	4.3	1.3E−04	–	Flightin
EZ974390	2.04	4.1	2.9E−04	Stretchin-isoform d	Stretchin-isoform d
EZ920519	2.00	4.0	1.3E−04	–	Actin, cytoplasmic
EZ973962	1.99	4.0	2.9E−04	Dimeric dihydrodiol dehydrogenase	Dimeric dihydrodiol dehydrogenase
EZ915590	1.98	3.9	1.6E−04	Venom allergen	A5R1: antigen 5 related protein 1
EZ915515	1.98	3.9	1.5E−04	–	CPCFC1: cuticular protein CPCFC family (CPCFC1)
EZ924022	1.95	3.9	1.5E−04	Pupal cuticle	Pupal cuticle
EZ916170	1.95	3.9	1.4E−04	Stretchin-isoform d	Stretchin-isoform d
EZ915759	1.88	3.7	2.9E−04	–	GSTS1: glutathione S-transferase sigma class 1
EZ976897	1.87	3.7	1.6E−04	Leucine-rich repeat-containing protein 20-like isoform 1	Leucine-rich repeat-containing protein 20-like isoform 1
EZ973739	1.87	3.6	1.6E−04	Lysozyme c-4	LYSC4: C-Type Lysozyme
EZ915209	1.85	3.6	3.6E−04	Glutathione S-transferase	GSTS1: glutathione S-transferase sigma class 1

The complete gene list is included as Additional file 1

**Table 8:** Cytochrome P450s and GSTs significantly over-transcribed in *Anopheles funestus* from DRC, when compared with the susceptible laboratory strain, FANG

Accession number	Name	Annotated description	Annotation against PEST	FC
EZ973782	Afun007663 (CYP6M7)	Cytochrome P450 6a8	CYP6M3	7.7
JX627312	CYP6P9b	Cytochrome P450	CYP6P3	3.0
EF152577	CYP6P13 (CYP6P9b)	Cytochrome P450	–	2.7
EZ975565	Afun009522	Cytochrome P450	–	2.6
EZ973498	Afun007369 (CYP6P9a)	Cytochrome P450	–	2.3
EU852645	CYP6P4b	Cytochrome P450	–	2.1
AY987359	CYP6P4	Cytochrome P450	–	2.0
EU852644	CYP6P4a	Cytochrome P450	–	2.0
EZ915759	cmb2_lrc578	Glutathione S-transferase	GSTs1	3.7
EZ915209	cmb2_lrc28	Glutathione S-transferase	GSTs1	3.6
EZ979266	Afun013481 (GSTe2)	Glutathione S-transferase	–	2.0

Criteria for significance were as follows: FC ≥ 2 and adjusted *p*-value ≤ 0.05

Interestingly, a number of immunity related genes were found to be under-transcribed. These included serine proteases, including *CLIPC7* and *CLIPB1*, a spondin, and trypsin and chymotrypsin like protein. In addition to these analyses, the microarray reporter sequences obtained here were blasted against *An. gambiae* (PEST) and *Aedes aegypti* genomes in order to supplement the annotations as the *An. funestus* reference genome is incomplete [63]. These annotations have been added to the Additional file 1.

Validation of microarray data was carried out on *CYP6P9b* and *CYP6M7*. The FC values obtained for these were in line with those obtained by microarray analyses. Specifically, *CYP6P9b* showed 5.8 (± 1.5)-fold higher expression relative to FANG, and *CYP6M7*, 6.3 (± 4.2)-fold higher expression than that of FANG. These values are based on the use of two RGs in each case.

## Discussion

This study aimed to characterize malaria vector profiles, insecticide resistance status and the mechanisms conferring resistance in the vectors in the DRC. These data are not only essential for insecticide resistance management in the region, but also contribute to the growing body of knowledge focussed on pyrethroid resistance.

Both of the surveys carried out in 2011 and 2012 identified the major vectors *An. funestus* and *An. gambiae s.s.*, as confirmed by PCR. The indoor house-spraying programme implemented after the initial field assessment in 2011 was based on the use of organophosphates and carbamates used in rotation. Both vector species were resistant to the Type II pyrethroid, deltamethrin. Of particular concern was the decrease in susceptibility to deltamethrin observed in *An. funestus* between 2011 and 2012—mortality dropped from 93% (2011) to 59% (2012). Such decreases in susceptibility can be due to over-use of a particular insecticide and in this instance, likely due to the widespread use of treated bed nets in the area, as well as the use of pyrethroids in both formal and informal agriculture. *Anopheles funestus* remained susceptible to all other insecticides tested, and critically, no changes in susceptibility to bendiocarb or the organophosphates were observed. In *An. gambiae*, the situation was more complex: mortality against DDT dropped from 60% (2011) to 15% (2012), and resistance to bendiocarb developed in the same period. In the absence of the use of DDT, the origin of resistance to the insecticide is not clear but may be related to cross-resistance conferred by pyrethroid resistance mechanisms such as *kdr* and the over-transcription of the cytochrome P450, *CYP6M2* [35]. The use of bendiocarb conferred rapid resistance to this insecticide, despite being used in rotation with the organophosphates. Such a situation emphasizes the need for regular resistance and vector monitoring so that adjustments to control programmes can be made timeously and accurately.

More than half of the *An. gambiae* samples assayed here were homozygous for the East African *kdr* mutation (L1014S), while 6% were heterozygous for this mutation. The West African *kdr* mutation (L1014F) was homozygous in 9% of the specimens, but no heterozygotes were found. In their study of *kdr* in the DRC, Basilua et al. [64] reported the presence of the L1014F (West African mutant) only, with the homozygous RR genotype predominating in 53% of the test population in 2009. Other studies of *kdr* in the central African region have reported the presence of both alleles in Cameroon [22] and the Congo [18]. The use of synergist assays indicates that *kdr* plays a limited role in pyrethroid resistance, but rather that cytochrome P450s are responsible. Carbamate resistance is most likely due to metabolic resistance and the absence of the *ace*-*1* ^*R*^ mutation supports this. Dieldrin is no longer used, but low levels of *rdl*-mutation were observed.

Further characterization of metabolic resistance mechanisms in both species were either through qPCR or microarray analysis. The use of microarray based studies and qPCR have provided relatively simple means to determine the identity of specific metabolic genes associated with resistance phenotypes. This is supported by the use of the synergist, PBO, which resulted in a reversion to 100% mosquito mortality in the presence of pyrethroids for *An. funestus* and 92% in *An. gambiae*. It is thought that the duplicated and highly polymorphic *CYP6P9a* and *CYP6P9b* genes [65, 66], along with *CYP6M7* are driving the spread of pyrethroid resistance northwards from Mozambique, Malawi and Zambia [41, 67]. Typically, both *CYP6P9a* and *CYP6P9b* are found to be over-transcribed in pyrethroid resistant *An. funestus* at much higher levels than those observed here [41] and this may reflect differing origins of resistance from resistant populations in southern Africa [68, 69], with decreasing over-transcription of these enzymes from Mozambique to Zambia [41, 68] to the DRC. A recent report by Riveron et al. [41] shows that both enzymes are able to confer resistance to pyrethroids independently and *CYP6P9b* is metabolically active against Type I and Type II pyrethroids. Similarly, *CYP6M7* (over-transcribed by 7.7-fold) is metabolically active against the pyrethroids [41]. These data expand the previously reported northern range of over-transcribed *CYP6P9a*, *CYP6P9b* and *CYP6M7* in *An. funestus* from Zambia to northeastern DRC.

*GSTe2* has been well documented in the context of DDT resistance and elevated levels of transcription have been reported in *An. funestus* [34], *An. gambiae* [70] and *Aedes aegypti* [71]. *GSTe2* has been closely linked to insecticide resistance in *An. funestus* from West Africa where elevated transcription, enhanced by a leucine to phenylalanine replacement (L119F), confers resistance to DDT and cross-resistance to pyrethroids [34, 72]. The presence of L119F should be assessed in subsequent work in the DRC in order to determine if this mutation is present in the region, however, the fact that full reversion to pyrethroid susceptibility in the presence of PBO would suggest a negligible role for this enzyme in conferring pyrethroid resistance.

In their study of the sigma class *GST 1* in *Apis cerana cerana*, Yan et al. [73] reported that the enzyme plays an important role in limiting oxidative damage and may play a role in detoxification of xenobiotics. Its specific function in *Anopheles* species is yet to be determined but the role of reducing oxidative damage is essential in protection against insecticides, particularly pyrethroids. Furthermore, the over-transcription of *GSTS1*-*2* has been found in the DDT and pyrethroid resistant phenotype of *An. arabiensis* [20] and pyrethroid resistant *An. gambiae* [74], and combined with the present data, suggest a role in insecticide resistance, even if only as a secondary function.

Cuticle thickening has been detected in a number of resistant mosquito species and enhances resistance by increasing the barrier against insecticides at the point of contact. Over-transcription of cuticle genes has been reported in *An. gambiae s.s*. [75] and *An. funestus* [76] and through the use of scanning electron microscopy [77], shown that the cuticle of pyrethroid resistant *An. funestus* was thicker than in susceptible mosquitoes. Larval and pupal cuticle-associated genes, along with a range of other cuticular genes were over-transcribed in a range of 2.1–8.2-fold.

Given the exceptionally high prevalence of malaria parasites in the vector populations in the area, and the fact that the data were generated from wild mosquitoes, it is interesting to note that a number of immune related genes were under-transcribed. Rivero et al. [78] proposed two explanations for this effect: 1) immunity may be compromized due to limitations in resources as the mosquito physiology is geared toward insecticide resistance; and 2) it is possible that insecticide resistance genes have a pleiotropic effect on genes associated with immunity. Similar outcomes have been observed in other insecticide resistant mosquito populations [79] and may be in response to the oxidative burden generated by P450 metabolism (as the prophenoloxidase cascade is also associated with oxidative stress) [80]. The CLIP proteins, along with trypsin and chymotrypsin like proteins have previously been implicated in mosquito immunity against *Plasmodium* [81], and recently RNAi mediated silencing of the serine protease, *ClipC9*, was shown to result in enhanced numbers of midgut oocysts [82].

Based on previous reports, a suite of ten detoxification enzyme genes were selected for assay by qPCR in *An. gambiae*. Such a strategy for testing metabolic resistance is better suited to an African context where expensive and highly technical assays are not always feasible due to lack of resources and funding. A suite of 10 genes was selected for testing metabolic resistance mechanisms in *An. gambiae*. The GST, *GSTS1*-*2*, showed the highest over-transcription with 31- and 7.6-fold increases in males and females respectively, followed by *GSTe2*, *TPX 2* and *CYP6M2*, the orthologue of *CYP6M7* in *An. funestus*.

A number of genes that were tested in *An. gambiae* showed only slight over-transcription despite having been significantly over-transcribed in other reports. This serves to highlight the fact that insecticide resistant populations of different *Anopheles* species and from different localities employ a multi-faceted system for protection. From this point of view, the testing of redox-associated transcripts is difficult. They play a critical role in resistance but as highlighted by Bonizzoni et al. [75], different enzymes are over-transcribed in different populations.

## Conclusion

To date, limited information is available on the molecular mechanisms of insecticide resistance in the DRC, a country with a severe malaria burden. In the north-eastern part of the country, high levels of pyrethroid resistance were present. Fortunately, organophosphates remain effective and this work emphasizes the need for continued surveillance, and avoidance of a one-size-fits-all approach to vector control and insecticide resistance management.

## Additional file

**Additional file 1.** Anopheles funestus annotations of the microarray reporter sequences.

## Abbreviations

*ace*-*1*^*R*^: acetylcholinesterase mutation
CYP: cytochrome P450
DDT: dichlorodiphenyltrichloroethane
DRC: Democratic Republic of Congo
ELISA: enzyme-linked immunosorbent assay
GABA: γ-amino butyric acid
GST: glutathione-s-transferase
IRS: indoor residual spraying
*kdr*: knockdown resistance
LLINs: long-lasting insecticide treated bed nets
PCR: polymerase chain reaction
PBO: piperonyl butoxide
qPCR: real-time quantitative PCR
RG: reference gene
RR: homozygous resistant
RS: heterozygous resistant
TPX: thioredoxin peroxidase
VGSC: voltage gated sodium channel
WHO: World Health Organization
